# Development of a novel Colorimetric Assay for the rapid diagnosis of Coronavirus disease 2019 from nasopharyngeal samples

**DOI:** 10.1038/s41598-024-53747-0

**Published:** 2024-05-27

**Authors:** Neda Sepahi, Sahar Samsami, Yaser Mansoori, Maryam Chenari, Negin Namavari, Ava Yazdanpanah, Abdolmajid Ghasemian, Zahra Montaseri, Mahboobeh Sharifzadeh, Razie Ranjbar, Sahar Namavari, Ali Ghanbariasad

**Affiliations:** 1 Noncommunicable Diseases Research Center, Fasa University of Medical Sciences, Fasa, Iran; 2 Student Research Committee, Fasa University of Medical Sciences, Fasa, Iran; 3 Department of Virology, School of Public Health, Tehran University of Medical Sciences, Tehran, Iran; 4 School of Medicine Grenada, St. George’s University, St. George’s, West Indies Grenada; 5 Department of Infectious Diseases, School of Medicine, Fasa University of Medical Science, Fasa, Iran; 6 Department of Medical Biotechnologies, School of Advanced Technologies, Fasa University of Medical Sciences, Avicenna Square, Fasa, Fars Islamic Republic of Iran

**Keywords:** Coronavirus disease 2019, Diagnosis, Real-Time PCR, AuNP-probe conjugate, Biotechnology, Cell biology, Microbiology

## Abstract

Emergence of Coronavirus disease 2019 (COVID-19) pandemic has posed a huge threat to public health. Rapid and reliable test to diagnose infected subjects is crucial for disease spread control. We developed a colorimetric test for COVID-19 detection using a Colorimetric Assay based on thiol-linked RNA modified gold nanoparticles (AuNPs) and oligonucleotide probes. This method was conducted on RNA from 200 pharyngeal swab samples initially tested by Real-Time polymerase chain reaction (RT-PCR) as gold standard. A specific oligonucleotide probe designed based on ORF1ab of COVID-19 was functionalized with AuNPs-probe conjugate. The exposure of AuNP-probe to isolated RNA samples was tested using hybridization. In this comparative study, the colorimetric functionalized AuNPs assay exhibited a detection limit of 25 copies/µL. It was higher in comparison to the RT-PCR method, which could only detect 15 copies/µL. The results demonstrated 100% specificity and 96% sensitivity for the developed method. Herein, we developed an incredibly rapid, simple and cost-effective Colorimetric Assay lasting approximately 30 min which could process considerably higher number of COVID-19 samples compared to the RT-PCR. This AuNP-probe conjugate colorimetric method could be considered the optimum alternatives for conventional diagnostic tools especially in over-populated and/or low-income countries.

## Introduction

Coronavirus disease 2019 (COVID-19) pandemic caused by the novel severe acute respiratory syndrome Coronavirus 2 (SARS-CoV-2) have left huge concern^1,2^. There is a widespread concern from the SARS-CoV-2 causing severe health issues due to its potential of infectivity and high rate of transmissibility in humans^2,3^. A number of vaccines (e.g., mRNA vaccines from Pfizer and Moderna) have been confirmed and administered in public^4^. However, their applications are in the initial stages, hence early diagnosis is crucial to control its spread^5^. The timely management and isolation of infected subjects are currently implemented based on time-consuming laboratory approaches^6^. The reverse transcription-polymerase chain reaction (RT-PCR)-based test has served for the viral RNA detection deemed as the gold standard technique for diagnosis of the COVID-19^7^. While RT-PCR offers acceptable sensitivity and reliability, it also comes with both costs and benefits. However, it has certain drawbacks, such as limited accessibility and affordability in many settings, particularly in less affluent countries. Additionally, it is mainly performed by well-trained staffs in a centralized laboratory/hospital in remote areas^7,8^. Although the lag between sampling and delivery is unpredictable on a large-scale for the pandemic, the average turnaround time for test results is estimated between 24 and 72 h worsen and prolonged when suspected samples need to be transported to an aforementioned laboratory at a distance. The resulting cost and turn-around-time of RT-PCR limit its feasibility for detection of large numbers of SARS-CoV-2 in a short time span^8–10^. Affordability, rapidity, convenience, portability, enough sensitivity and specificity of a method crucially determine the efficiency of diagnosis for such a threatening fulminant agent^11^. Nanotechnology-based colorimetric bioassays have advantage of convenience for designing Colorimetric Assay because of their simplicity, visual output, and no requirement for complex facilities^12,13^. Having unique optical properties, the particular nanoparticles can create obvious color change visualized by naked eye or quantitative measurement by simple optical detector^14^. Gold nanoparticles (AuNPs) are among the most applicable nanomaterials for biomolecule detection due to their unique optical characteristics, large surface area and ability of functionalizing with various biomolecules^15,16^, being appropriate for virus surveillance as detection probes^17^. In this research, we aimed to develop Colorimetric Assay based on thiol-linked RNA modified AuNPs relying on hybridization of target SARS-COV-2 RNA with complementary oligonucleotide probes.

## Methods and materials

### Ethics statement

Nasopharyngeal swap samples (n = 200) were collected from Fasa University of Medical Science Hospital, Iran (stored in Virus transport medium (VTM)) in accordance with the approved guidelines and relevant regulations. All subjects provided their written informed consent prior to participating in the study. The Ethics Committee of Fasa University of Medical Science approved all experimental procedures (IR.FUMS.REC.1399.041). All experiments were performed in accordance with the approved guidelines.

### RNA extraction

The COVID-19 genomic RNA was extracted from 160 µL of suspected cases’ nasopharyngeal swabs using QIAamp Viral RNA Kits (Qiagen, Hilden, Germany) in accordance with the manufacturer’s protocol. After elution in RNase-free water, RNA samples were stored in aliquots of 20 µL at – 80 °C until required.

### Real-time PCR assay

In order to detect SARS-COV-2 in suspected cases, a commercial RT-PCR kit (nCoV real-time detection kit, Sansure Biotech) was used. RT-PCR was carried out according to the manufacturers' instructions. A 50 µL total reaction mixture contained 20 μL of RNA template, 26 μL nCov-PCR Mix and 4 μL nCov-PCR-Enzyme Mix. The RT-PCR assay was performed at 50 °C for 30 min for reverse transcription, followed by 95 °C for 1 min and then 45 cycles of 95 °C for 15 s, 60 °C for 30 s recruiting BioRad system (CFX96 Touch Real-Time PCR Detection Systems). RT-PCR with CT value of > 39 were considered as negative result.

### Synthesis and characterization of AuNPs

Citrate-capped AuNPs with an average diameter of approximately 20 nm were successfully synthesized using a well-established protocol^18,19^. Having been prepared, a 50 mL solution containing 0.7 mM of HAuCl_4_ was heated under reflux. Under vigorous stirring, 5 mL of 25.9 mM trisodium citrate was quickly added to the solution once it reached the boiling point. Afterwards, reflux was continued for 30 min. The solution color changed into deep-red that confirmed the formation of AuNPs and left in ambient temperature. Final concentration of the particle was estimated at 10 nM based on Beer’s law applying extinction coefficient of AuNPs 20 nm at 524 nm^18,19^.The synthesized AuNP solution was stored in a dark and cool place (4 °C) for further use. The particle length distribution (SPAN) and mean diameter values of particle size (ps) of the AuNPs had been determined the usage of dynamic light scattering (DLS) (scatteroscope, okay-ONE NANO. LTD, Korea). Morphology, size and the characterization of the particles were investigated using Transmission Electron Microscope (TEM) (Libra 120 TEM) considering accelerating voltage of 400 kV.

### Probe design

A 20-base synthetic oligonucleotide probe was designed according to the deposited sequence in GenBank (NC_045512) revolving around ORF1ab of COVID-19 as target gene. The probe has been designed to specifically detect COVID-19 agent. Basic Local Alignment Tool (BLAST) was used by the employment of the National Center for Biotechnology Information (NCBI) database. Next, a thiol group was added to the 5′ end of probe, facilitating its conjugation with the colloidal AuNPs. The 5′Thiol-CTGCGGTATGTGGAAAGGTTATG-3′ was purchased from Bioneer company (South Korea).

### Functionalization of AuNPs with reporter probe

In order to activate the thiolated probe, the thiol groups were reduced by re-suspending it with 20 µL of Tris (2-carboxyethyl) phosphine (TCEP) (0.2 M/L) at room temperature for an hour^20^. The next step in the process was probe integration with AuNPs as previously described^20^. Then, 1000 µL AuNPs of mixture and 20 µL thiol modified oligonucleotide were vortexed overnight at room temperature in dark conditions. Before adding the SDS solution, the concentration of the solution was 9 mmol/L in a sodium phosphate buffer with a pH of 7. The addition of the SDS solution at a concentration of 0.1% was intended to prevent aggregation.

The volume of salting buffer (2 M NaCl in 10 mM PBS PH 7) which was divided into six doses was added to the solution gradually at room temperature for 2 days. To maximize DNA packaging, a 30-s sonication of particles was required after each salt addition.

After being centrifuged at 16,000 g for 30 min at 4 °C, the precipitate was washed with 500 µL and resuspended in 200 µL of washing buffer (NaCl 1.5 mM, 0.1% SDS in PBS Buffer) before stored at 4 °C in dark place. The DNA-functionalized AuNPs and the pure AuNPs suspension should show the same color. To confirm the conjugation of AuNPs-probe, the salt-induced aggregation test was conducted. In this test, 5 µL of probes and AuNPs were mixed separately with 1 µL of MgCl2 (5 mM) for 10 min, without any observed color change^21,22^.

### AuNPs Colorimetric Assay

The Colorimetric Assays were implemented in a total volume of 20 µL for 30 min comprising of a process by which COVID-19 genomic RNA could be detected. The procedure commenced by heating 10 µL of target DNA sample to 95 °C for 5 min, followed by immediate ice cooling of the test tubes. Prior to being incubated at 63 °C for 10 min, 4 µL of the AuNPs-probe was added and mixed with the solution. The final phase of the process occurred 10 min later, involving the addition of 5 µL of 0.2 N HCl to the reaction. The same protocol was implemented for preparing a blank control without target sequence. After being allowed to remain at room temperature for 5 min, the mixtures developed their desired color, which could be detected by the naked eye and confirmed through an absorption spectrum^21,22^.

### Minimum detection limit (MDL)

To assess the minimum detection limit and quantification of COVID-19 genomic RNA, a serial dilution of a positive control (200,000 copies per µL) available in the Coronavirus diagnostic kit (QIAamp Viral RNA Kits) was prepared and examined. Then, standard curve was obtained from the 10 COVID-19 positive samples. RT-PCR was performed on the samples and the number of copies was determined based on the standard curve. In the following, the two-fold serial dilution from mixed samples was performed. The copy numbers of the 10 samples were 50,000 copy number/µL.

### Clinical sample analysis

A total of 200 samples were collected from sex matched patients with mean age of 52 years old and analyzed by RT-PCR, 100 of them were confirmed as COVID-19 infected patients. Those RT-PCR negative individuals had not any contact with COVID-19 patients and also had not any migration to suspected places. To ensure that they were not infected, individuals were selected from groups who intended to travel abroad or join military service. When selecting patients, it was ensured that apart from the positive test of RT-PCR, all of them exhibited a range of COVID-19 clinical symptoms, varying from mild to severe. Also we performed the test on 20 influenza-positive samples and 20 RSV positive samples and all of which were detected as negative.

### Ethics approval and consent to participate

The authors confirm that all methods were carried out in accordance with relevant guidelines and regulations. All experimental protocols were approved by Fasa University of Medical Sciences. The informed consent was obtained from all subjects and/or their legal guardian(s).

## Results

### Characterization of AuNPs

**Figure 1:**
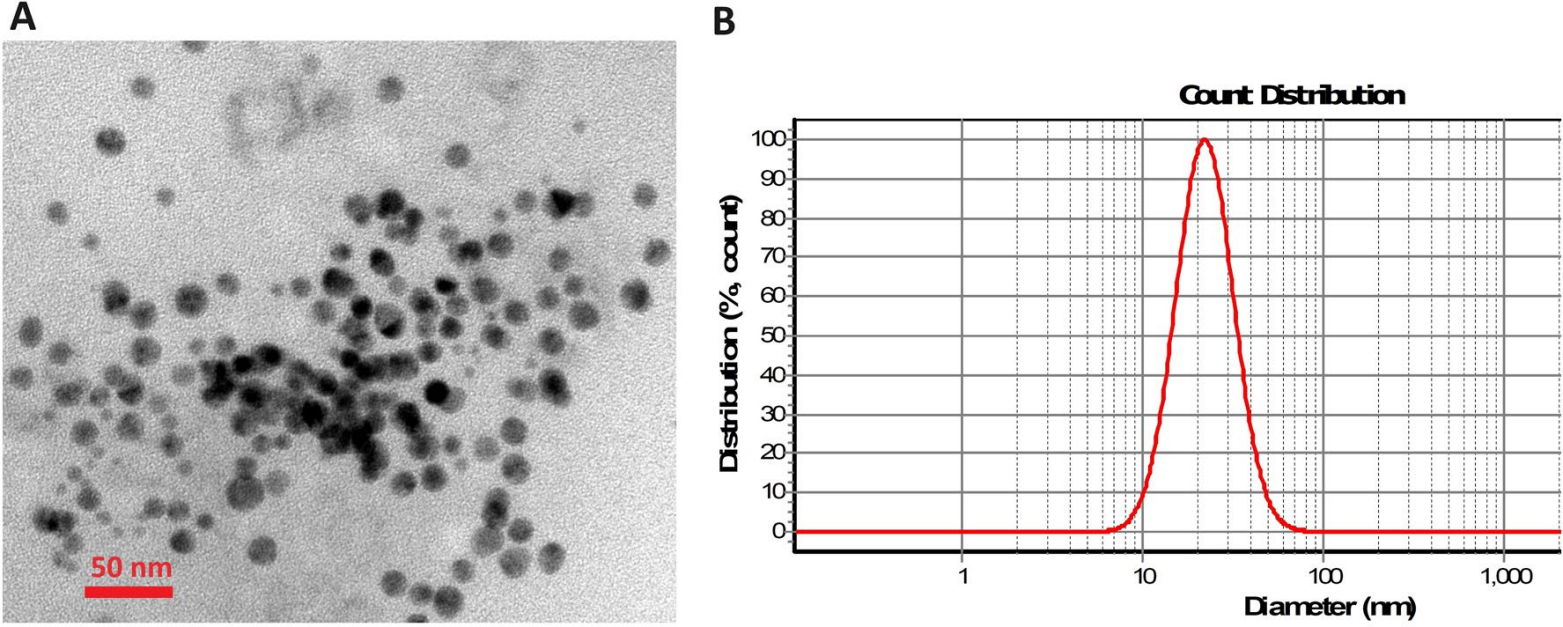
(**A**) The TEM image of synthesized AuNPs with the mean size of 20 nm, (**B**): the size distributions of AuNPs via DLS analysis.

The transmission electron microscopy (TEM) image of the synthesized AuNPs with a mean of diameter 20 nm has been illustrated in Fig. 1A. The single particles size diameter included 20 ± 5 nm using DLS analysis that shown in Fig. 1B.

A red solution was attained for the AuNPs mixture. The functionalization of AuNPs with probe was indicated by giving more intense color than that of the synthesized AuNPs which was darker following increased level of concentration during functionalization process.

**Figure 2:**
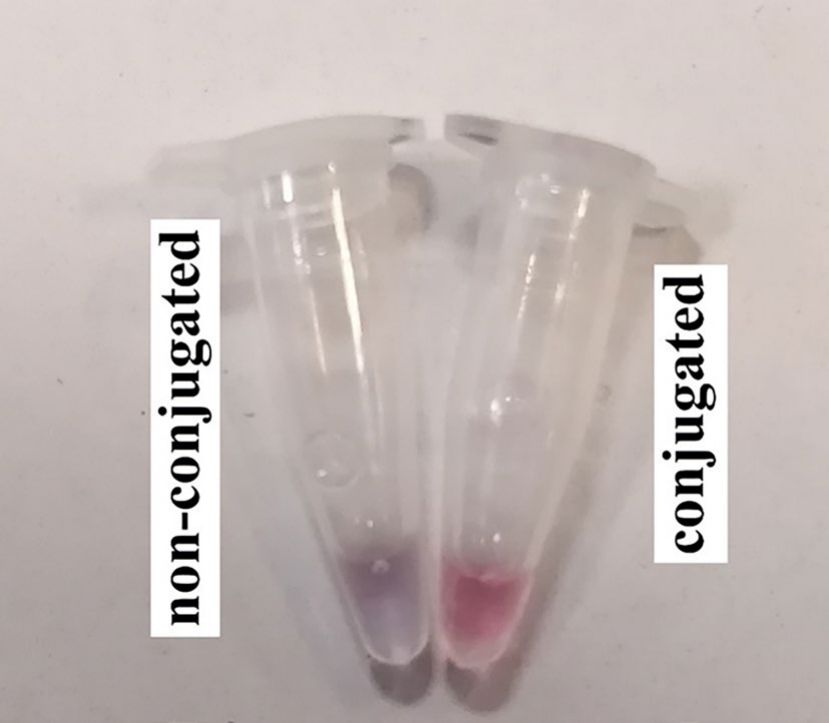
Illustration of conjugated and non-conjugated AuNPs.

The salt-induced aggregation test (resistance test) was applied in order to determine the strength of the conjugation of the probes with AuNPs. The mixture with 1 µL of MgCl_2_ (5 mM) in two separated microtubes, 5 µL of nanoprobes and non-conjugated AuNPs solutions were compared. The AuNPs showed resistance to MgCl_2_ induced aggregation resulting in purple color (Fig. 2).

In acidic conditions, the AuNPs-probe solutions color was altered from red to blue purple. The assessment of AuNPs-probe distinguishability for detecting sequence of SARS-COV-2 complementary RNA was done with the exposure of non-complementary and different amounts of complementary sequences. Double stranded acid nucleic which was evolved due to hybridization of AuNPs-probe with complementary sequence was able to improve the AuNPs-probe stability against aggregation, remaining its color unassailable. On the other hand, the other condition in which the solution turned into purple, AuNPs-probe was of high probability to be aggregated following addition of the 0.2 N HCl.

### Limit of detection

**Figure 3:**
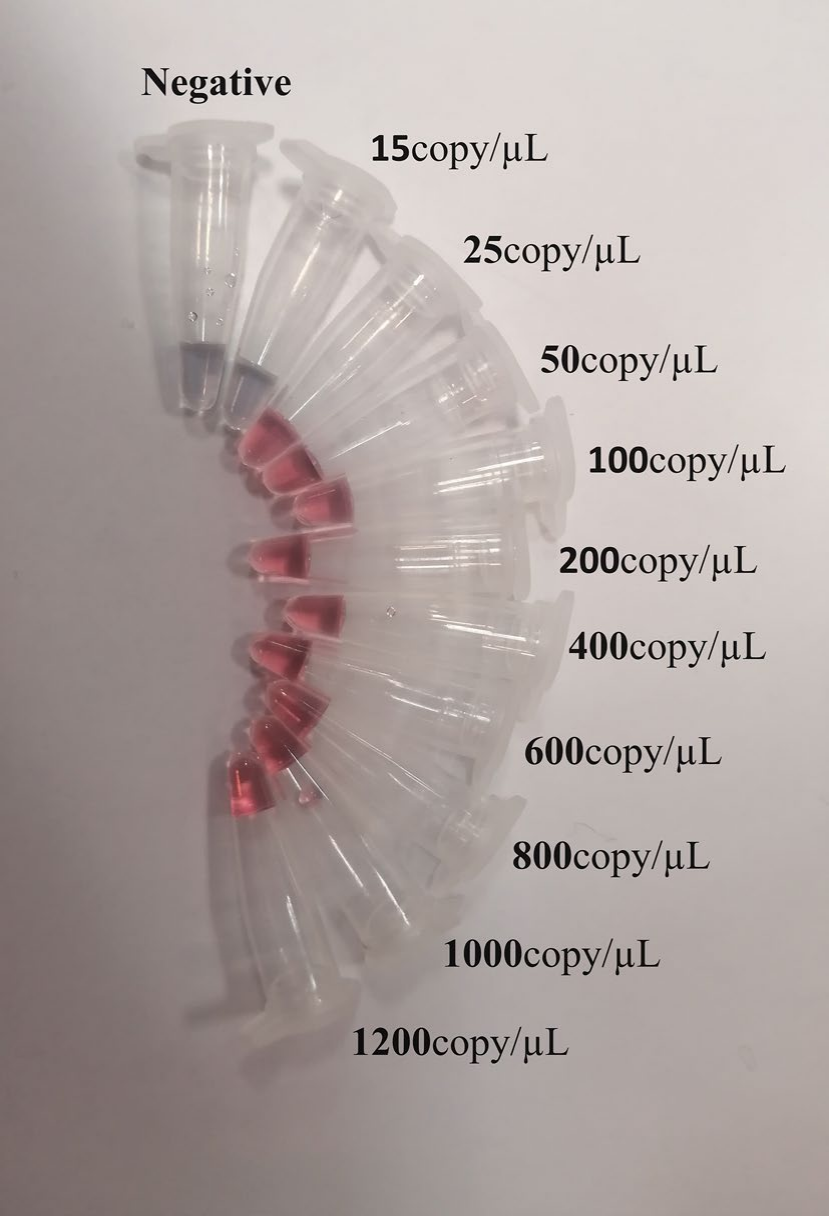
The color change of different volume extraction of positive and negative samples after exposure to 0.2 N HCl. The red color indicates dispersed nanoparticles whereas the purple color indicates aggregated nanoparticles.

To assess the AuNPs-probe hybridization effectiveness for limit of detection, different volumes of extracted genome of positive (1200 copy/µL, 1000 copy/µL, 800 copy/µL, 600 copy/µL, 400 copy/µL, 200 copy/µL, 100 copy/µL, 50 copy/µL, 25 copy/µL and 15 copy/µL) and negative samples were prepared. Acid addition formed the color of different amount of positive and negative samples by exposure with AuNPs-probe as represented in Fig. 3.

**Figure 4:**
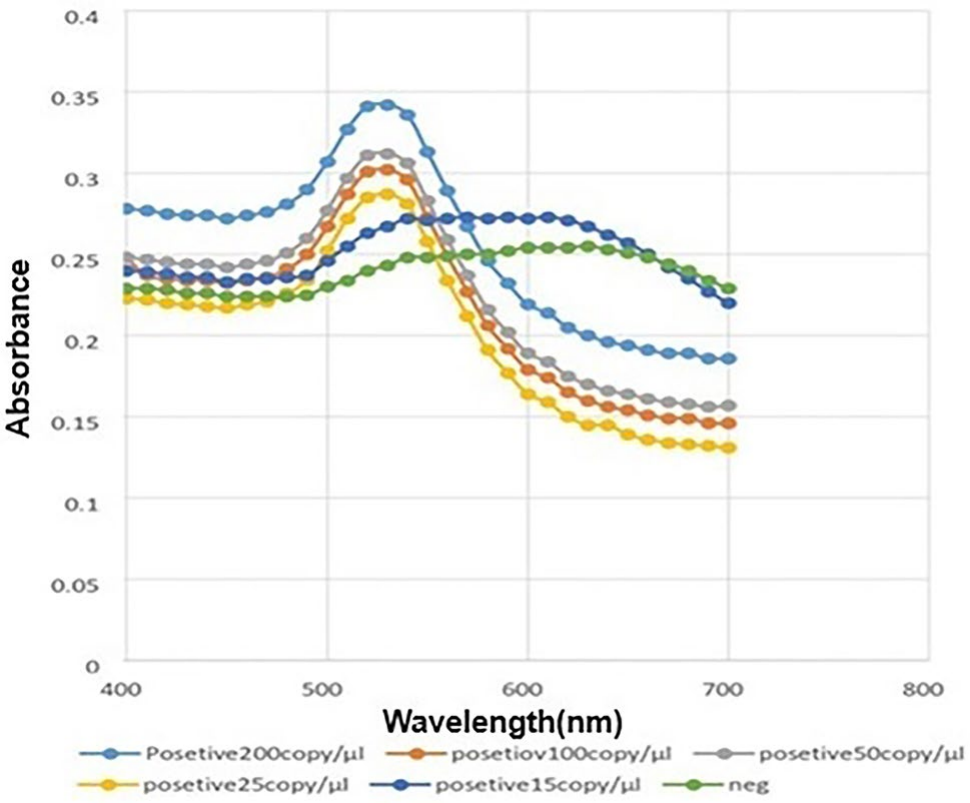
UV–vis spectrum illustrates the absorption wavelengths. UV-spectra of positive samples which each color indicate a distinct volume of extracted RNA (absorption peak approximately 550 nm) and negative samples showing in green and dark blue lines (absorption peak around 620 nm).

Images showed the solutions including 25 copy number per µL of RNA of Coronavirus remained red, while the negative samples color turned into purple. UV–vis spectroscopy and DLS analysis were performed on both single AuNPs and AuNPs conjugated with probes. The analysis revealed a particle size diameter of 20 ± 5 nm for the single particles, as determined using DLS. UV–vis spectra of specimen containing 200 copy/µL, 100 copy/µL, 50 copy/µL, 25 copy/µL, and 15 copy/µL COVID-19 genome presented a maximum peak at 528 nm and regarding negative samples, a wide absorbance spectrum was around 620 nm (Fig. 4). Hence, the confirmation of hybridization of AuNPs-probe with the presence of the viral acid nucleic was achieved by visual and spectrophotometry ways.

### Clinical samples

**Figure 5:**
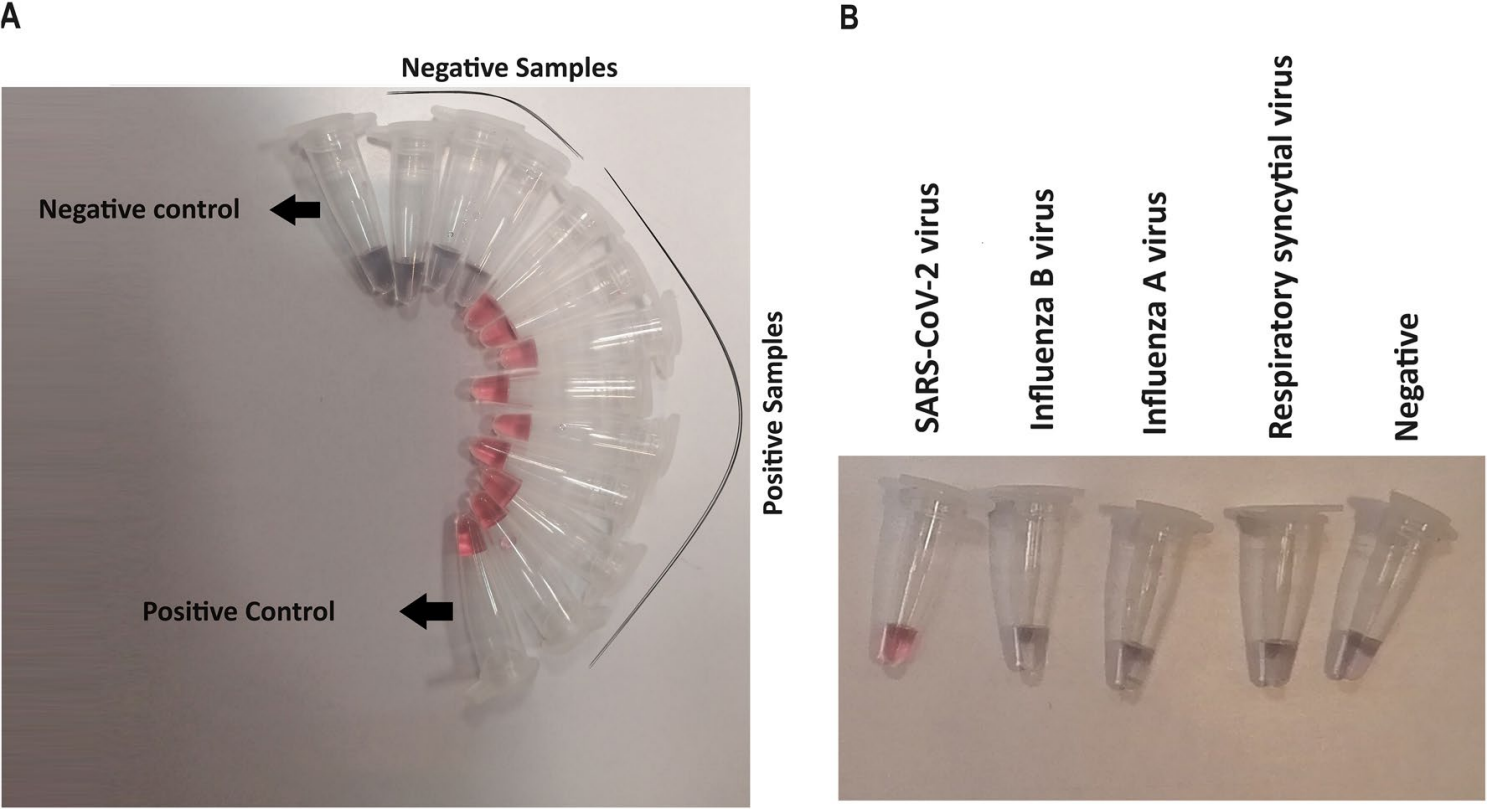
(**A**) Evaluation of the COVID-19 Colorimetric Assay diagnostic test applicability using infected and non-infected cases’ samples. (**B**) The test result on other viral samples was negative.

To validate the performance of the Colorimetric Assay in diagnosing COVID-19, 200 clinical samples (100 positives and 100 negatives) previously confirmed by the RT-PCR technique as a gold standard were utilized and their threshold cycles were represented in the Table 1. In order to determine the assay detection limit, a two-fold serial dilution of isolated SARS-COV-2 RNA was prepared. Based on the results, this assay was able to detect as few as 25 copies/µL. On the other hand, the RT-PCR with limit of detection (LOD) of 15 copies/µL indicating higher sensitivity. A total of 96 of 100 COVID-19 patients diagnosed by the RT- PCR remained unchanged in terms of color and identified positive by our studied assay showing 96% sensitivity. The four false negative samples with CT values between 36 and 39 contain too low viral load to be identified as positive cases. However, all the RT-PCR confirmed negative samples gave the same results by the assay; accordingly, the specificity of this approach was 100%. The testing results on COVID-19 infected patients has been shown in Fig. 5A. Also for selectivity, the test was performed on other virus samples as shown in Fig. 5B.

**Table 1:** Patients’ numbers and CT values of RT- PCR.

Number of patients	Cycle of threshold
20	10–15
20	16–20
20	21–25
15	26–30
12	31–35
13	36–39

## Discussion

COVID-19 pandemic respiratory disease has caused a huge global threat on account of its rapid spread and high fatalness^1^. This concern is deemed to be of acuteness inasmuch as statistics indicated the number of confirmed cases of COVID-19 had exceeded 702,141,250 cases with 6,971,929 deaths throughout the world^2^. There are various methods for the COVID-19 diagnosis with some limitations. For instance, high costs, susceptibility to contamination, complexity, limited information (RT-PCR and next generation sequencing), contamination risk and primer design challenges (LAMP), specificity concerns, overuse and unnecessary imaging, radiation exposure and resource-intensity (CT scan), false negatives and positives, inability to distinguish between active and past infections, timing of detection, limited information on immunity (serology) include main drawbacks of these conventional approaches^23–28^. A special tool for mass screening, if not taken into consideration in terms of accuracy, can pose a dire threat to public health on world scale especially on countries with low or mild incomes, densely populated or remote regions which could be more vulnerable^29^. This study established one of the most rapid, simple, and cost-effective assays for SARS-CoV-2 detection by a Colorimetric Assay based on thiol-linked RNA modified AuNPs. The test results can be directly judged through the color change by naked eye without use of special facilities and the reaction time takes only 30 min. At a preliminary stage of this work, a specific probe for SARS-CoV-2 was to develop. Our accurate designed probe played an indispensable role to reach this assay’s specificity to the highest degree showing no false positive results. Our results indicated that whereas the Colorimetric Assay using the ORF1ab probe was not sensitive enough to replace RT-qPCR in all applications when the viral load is too low, it can hold promise as a method for testing a great deal of samples within a short time which would be one of the most appropriate and fastest screening tools in low-resource settings.

Direct detection of unamplified genome can be done by functionalizing AuNPs with specific thiolated probes^30^. Optical characteristics of AuNPs lead to being utilized for colorimetric detection which is fast and convenient with low cost^31^, hence, it can be considered one of the optimum alternatives for conventional diagnostic tools. In this study, we developed a simple approach based on a non-cross-linking hybridization method, where the aggregation of AuNPs-probe was induced by an increasing acid concentration. Once AuNPs are modified with single stranded DNA, the dispersion of the ssDNA–AuNP complex is stable in a solution of high ionic strength due to inter-particle electrostatic and steric repulsion between the ssDNA-modified AuNPs (ssDNA-AuNPs)^32^. The difference in optical features of aggregated and dispersed AuNPs is the main reason of this colorimetric hybridization change. The mixture of AuNPs and AuNP-probe are red with a relatively narrow surface plasmon absorption band at 520 nm in UV–vis spectrum^31^, while, the surface plasmon resonance is changed from 520 to 550 nm without the presence of any genomic target material^33^. In this study, the stage of denaturation of target RNA occurred at 95 °C for 5 min following immediate ice cooling, adding the AuNPs-probe and annealing at 63 °C for 10 min; once HCl was added, the results was visualized within 1 min by the naked eye. Turning into purple, clinical samples were free from COVID-19 RNA, vice versa, it was considered positive when its color remained unchanged. Furthermore, omission of nucleic acid amplification turned out to be more advantageous in a considerable number of cases as it can be done with faster time to results by simple and low-cost equipment using a simple water bath or a heating block rather than thermocycler. The whole reaction time was at most 30 min being 4–sixfold faster than the time required for RT-PCR. Moreover, the test was considerably easy to be trained within a few hours without need of experienced molecular biologists. Contrary to high cost of RT-PCR test which costs 14 US$ per reaction, it offers highly cost-effective assay with 9 US$ for each sample.

Another criteria which should be considered for devising a viral diagnostic tool is conserved RNA elements^34^. In SARS-COV-2, the entire region of ORF playing significant role in vial life cycle is flanked by 50 and 30 untranslated regions (UTRs) and has been found in other members of Coronavirus family to contain conserved RNA structures^35,36^. The enzymatic proteins are mainly encoded by ORF1ab. Other proteins encoded by ORF1ab include surface glycoprotein (S), small envelope proteins (E), matrix proteins (M), nucleocapsid proteins (N) and several non-structural proteins^37^. The sensitivity of *RdRP* (RNA-dependent RNA polymerase) gene responsible for ORF1ab region, was considerably greater than N gene in a study of Moitra et al.^38^. The limit of detection for these genes were 3.6 and 8.3, respectively, indicating the former region could be more appropriate option for a Colorimetric Assay. This colorimetric detection of SARS-CoV-2 using antisense oligonucleotide capped plasmonic gold nanoparticles is able to get the result within 10 min^38^. In the current study, UV–vis spectra of positive samples with various volume: 1000 copy/mL = 34 ng, 800 copy/mL = 30, 400 copy/mL = 31, 200 copy/mL = 28 ng COVID-19 genome showed a maximum peak at 528 nm and negative samples made a wide absorbance spectrum up to 620 nm. Turning into clinical samples, we considered RT-PCR test as a gold standard and evaluated the obtained results of the assay compared to the RT-PCR results. There was no false positive finding, ensuring 100% specificity of the assay. However, with four false negative results, the protocol sensitivity was decreased to 96%. Limit of detection was 25 copies/μL which was 1.5 time higher than that of RT-PCR (15 copies/μL). The result of this research has certain aspects in common with Alafeef et al.’s protocol with 100% and 96.6% specificity and sensitivity, respectively, with a previous detection limit of 10 copies/μL. Their RNA-extraction-free nano-amplified colorimetric test uses antisense oligonucleotides (ASOs)-capped plasmonic AuNPs and as a colorimetric reporter was able to detect the current imposed virus in less than 1 h^39^. Another simple and rapid Colorimetric Assay for detecting MERS-CoV detected by the naked eye without any sophisticated equipment within 10 min was developed by Kim and colleagues. That study proposed a colorimetric detection tool using disulfide bonds formed by hybridizing with thiolated probes and a target. This method inhibited the aggregation of AuNPs by salt and decreased the diagnosis of MERS detection limit of 1 pmol/μL^40^.

Recently, rapid colorimetric amplification assays such as loop-mediated isothermal (lamp), nanosensors (oligonucleotide capped) and antisense oligonucleotides (ASOs) have been applied for SARSCoV-2 identification with various sensitivity values^38,41,42^. The thiol-modified ASOs specific for N gene inferred detection limit of 0.18 ng/μL of RNA^38^. Another Colorimetric Assay using AuNPs directly detected SARS-CoV-2 RNA from saliva and nasopharyngeal swab samples with detection limit of 2.1 × 10^5^ copies /mL^43^.

The analytical sensitivity of the Colorimetric Assay can be improved by targeting of multiple genetic regions within ORF1ab gene sequence simultaneously. Nevertheless, the merits would seem to overshadow the demerits of the approach feasibility. This test was inexpensive and specific, but less sensitive compared to the RT-PCR. However, higher rapidity for public detection makes it applicable more in many developing countries and resource-free settings.

## Conclusion

Mass detection is of utmost importance to decelerate the transmissibility of the life-threatening virus, SARS-COV-2, particularly in majority of third world countries in which sheer cost of current diagnostic tools such as RT-PCR is beyond the financial support and also populated and/or remote regions require cost-effective, accurate and rapid diagnostic test. To address this predicament, our research group is the first one introducing a simple, specific, sensitive and rapid Colorimetric Assay using AuNPs by targeting ORF1ab of SARS-COV-2. This affordable and portable developed tool would diagnose a huge number of samples in a short period of time without foisting any limitation due to location, well-trained technicians or sophisticated equipment. The results are visible to the naked eye just by 30 min and can be interpreted only by color change. In addition, the cost and time of sample transporting to core laboratories would be greatly saved. This system failed to detect only four RT- PCR-positive samples when their CT values were above 35. Additionally, the specificity was 100% with no false positive.

## Data Availability

The datasets used and/or analyzed during the current study available from the corresponding author on reasonable request.

## References

[1] Wang C (2020). A novel Coronavirus outbreak of global health concern. The Lancet.

[2] Vakil, M. K. *et al.* Individual genetic variability mainly of Proinflammatory cytokines, cytokine receptors, and toll‐like receptors dictates pathophysiology of COVID‐19 disease. *J. Med. Virol.* **94**(9), 4088–4096 (2022). 10.1002/jmv.27849 PMC9348290 35538614

[3] Thompson D, Lei Y (2020). Mini review: Recent progress in RT-LAMP enabled COVID-19 detection. Sens. Actuator. Rep..

[4] Tumban E (2021). Lead SARS-CoV-2 candidate vaccines: Expectations from phase III trials and recommendations post-vaccine approval. Viruses.

[5] Lee D, Lee J (2020). Testing on the move: South Korea's rapid response to the COVID-19 pandemic. Transport. Res. Interdiscipl. Perspect..

[6] Miripour ZS (2020). Real-time diagnosis of reactive oxygen species (ROS) in fresh sputum by electrochemical tracing; correlation between COVID-19 and viral-induced ROS in lung/respiratory epithelium during this pandemic. Biosens. Bioelectron..

[7] Nopsopon T (2020). COVID-19 antibody in Thai community hospitals. medRxiv.

[8] Sharfstein JM, Becker SJ, Mello MM (2020). Diagnostic testing for the novel Coronavirus. JAMA.

[9] Pfefferle S (2020). Evaluation of a quantitative RT-PCR assay for the detection of the emerging Coronavirus SARS-CoV-2 using a high throughput system. Eurosurveillance.

[10] Kang S (2020). Recent progress in understanding 2019 novel Coronavirus (SARS-CoV-2) associated with human respiratory disease: Detection, mechanisms and treatment. Int. J. Antimicrob. Agents.

[11] Pokharel G (2017). Reaching adolescents with health services in Nepal. Bull. World Health Organ.

[12] Pan D (2014). Multicolor computed tomographic molecular imaging with noncrystalline high-metal-density nanobeacons. Contrast Media Mol. Imaging.

[13] Pan D (2010). Near infrared photoacoustic detection of sentinel lymph nodes with gold nanobeacons. Biomaterials.

[14] Zhao VXT (2020). Colorimetric biosensors for point-of-care virus detections. Mater. Sci. Energy Technol..

[15] Bakthavathsalam P, Rajendran VK, Mohammed JAB (2012). A direct detection of *Escherichia*
*coli* genomic DNA using gold nanoprobes. J. Nanobiotechnol..

[16] Huang X (2007). Gold nanoparticles: Interesting optical properties and recent applications in cancer diagnostics and therapy. Nanomedicine.

[17] Mokhtarzadeh A (2017). Nanomaterial-based biosensors for detection of pathogenic virus. Trends Analyt. Chem..

[18] Andreadou M (2014). A novel non-amplification assay for the detection of *Leishmania* spp. in clinical samples using gold nanoparticles. J. Microbiol. Methods.

[19] Sattarahmady N, Kayani Z, Heli H (2015). Highly simple and visual colorimetric detection of *Brucella melitensis* genomic DNA in clinical samples based on gold nanoparticles. J. Iran. Chem. Soc..

[20] Zou L, Li X, Lai Y (2021). Colorimetric aptasensor for sensitive detection of kanamycin based on target-triggered catalytic hairpin assembly amplification and DNA-gold nanoparticle probes. Microchem. J..

[21] He Y (2022). The application of DNA-HRP functionalized AuNP probes in colorimetric detection of citrus-associated Alternaria genes. Talanta.

[22] Elahi N (2019). A fluorescence Nano-biosensors immobilization on Iron (MNPs) and gold (AuNPs) nanoparticles for detection of *Shigella* spp.. Mater. Sci. Eng. C Mater. Biol. Appl..

[23] Halder A, Datta B (2021). COVID-19 detection from lung CT-scan images using transfer learning approach. Mach. Learn.: Sci. Technol..

[24] Axiaq A (2021). The role of computed tomography scan in the diagnosis of COVID-19 pneumonia. Curr. Opin. Pulmon. Med..

[25] Das D, Lin C-W, Chuang H-S (2022). LAMP-based point-of-care biosensors for rapid pathogen detection. Biosensors.

[26] Teymouri M (2021). Recent advances and challenges of RT-PCR tests for the diagnosis of COVID-19. Pathol.-Res. Pract..

[27] Vandenberg O (2021). Considerations for diagnostic COVID-19 tests. Nat. Rev. Microbiol..

[28] Peeling RW (2020). Serology testing in the COVID-19 pandemic response. Lancet Infect. Dis..

[29] Wolff D (2021). Risk factors for Covid-19 severity and fatality: A structured literature review. Infection.

[30] Verma MS (2015). Colorimetric biosensing of pathogens using gold nanoparticles. Biotechnol. Adv..

[31] Wang W (2008). Aptamer biosensor for protein detection using gold nanoparticles. Anal. Biochem..

[32] Kanayama N (2016). Terminal-specific interaction between double-stranded DNA layers: Colloidal dispersion behavior and surface force. Langmuir.

[33] Sattarahmady N (2015). Gold nanoparticles biosensor of *Brucella* spp. genomic DNA: Visual and spectrophotometric detections. Biochem. Eng. J..

[34] Spurgers KB (2008). Oligonucleotide antiviral therapeutics: Antisense and RNA interference for highly pathogenic RNA viruses. Antiviral Res..

[35] Madhugiri R (2018). Structural and functional conservation of cis-acting RNA elements in Coronavirus 5'-terminal genome regions. Virology.

[36] Chen C (2013). Dynamics of translation by single ribosomes through mRNA secondary structures. Nat. Struct. Mol. Biol..

[37] Lu R (2020). Genomic characterisation and epidemiology of 2019 novel Coronavirus: Implications for virus origins and receptor binding. The Lancet.

[38] Moitra P (2020). Selective naked-eye detection of SARS-CoV-2 mediated by N gene targeted antisense oligonucleotide capped plasmonic nanoparticles. ACS Nano.

[39] Alafeef M (2021). RNA-extraction-free nano-amplified colorimetric test for point-of-care clinical diagnosis of COVID-19. Nat. Protocols.

[40] Kim H (2019). Development of label-free Colorimetric Assay for MERS-CoV using gold nanoparticles. ACS Sens..

[41] Derin D (2023). Designing of rapid assay for the detection of RdRp/Orf1ab specific to SARS-CoV-2. J. Virol. Methods.

[42] Szobi A (2023). Vivid COVID-19 LAMP is an ultrasensitive, quadruplexed test using LNA-modified primers and a zinc ion and 5-Br-PAPS colorimetric detection system. Commun. Biol..

[43] Armesto M (2023). Validation of rapid and economic colorimetric nanoparticle assay for SARS-CoV-2 RNA detection in saliva and nasopharyngeal swabs. Biosensors.

